# The Hippo pathway is controlled by Angiotensin II signaling and its reactivation induces apoptosis in podocytes

**DOI:** 10.1038/cddis.2014.476

**Published:** 2014-11-13

**Authors:** D O Wennmann, B Vollenbröker, A K Eckart, J Bonse, F Erdmann, D A Wolters, L K Schenk, U Schulze, J Kremerskothen, T Weide, H Pavenstädt

**Affiliations:** 1 Internal Medicine D, Department of Nephrology, Hypertension and Rheumatology, University Hospital Muenster, Muenster, Germany; 2 Institute of Physiology I, Westfaelische Wilhelms-University, Muenster, Germany; 3 Analytical Chemistry NC4/72, Biomolecular Mass Spectrometry/Proteincenter, Ruhr-University Bochum, Muenster, Germany

## Abstract

The Hippo pathway fulfills a crucial function in controlling the balance between proliferation, differentiation and apoptosis in cells. Recent studies showed that G protein-coupled receptors (GPCRs) serve as upstream regulators of Hippo signaling, that either activate or inactivate the Hippo pathway via the large tumor suppressor kinase (LATS) and its substrate, the co-transcription factor Yes-associated protein (YAP). In this study, we focused on the Angiotensin II type 1 receptor (AT1R), which belongs to the GPCR family and has an essential role in the control of blood pressure and water homeostasis. We found that Angiotensin II (Ang II) inactivates the pathway by decreasing the activity of LATS kinase; therefore, leading to an enhanced nuclear shuttling of unphosphorylated YAP in HEK293T cells. This shuttling of YAP is actin-dependent as disruption of the actin cytoskeleton inhibited dephosphorylation of LATS and YAP. Interestingly, in contrast to HEK293T cells, podocytes, which are a crucial component of the glomerular filtration barrier, display a predominant nuclear YAP localization *in vivo* and *in vitro*. Moreover, stimulation with Ang II did not alter Hippo pathway activity in podocytes, which show a deactivated pathway. Reactivation of the LATS kinase activity in podocytes resulted in an increased cytoplasmic YAP localization accompanied by a strong induction of apoptosis. Thus, our work indicates that the control of LATS activation and subsequent YAP localization is important for podocyte homeostasis and survival.

The Hippo signaling pathway controls organ size by mediating the balance between proliferation, differentiation and apoptosis in cells.^1, 2^ Previous studies elucidated a high degree of evolutionary conservation for the Hippo pathway and showed that most of its components – initially identified in *Drosophila –* have functional homologs in the mammalian system.^1, 3, 4^ The core components of the Hippo pathway are the co-transcription factor Yes-associated protein (YAP) in mammals and Yorkie in *Drosophila*. The activity of YAP is controlled by the large tumor suppressor kinase (LATS), which phosphorylates and thereby inactivates YAP.^5^ This phosphorylation of YAP at Serine 127 (S127) avoids its nuclear shuttling and inhibits expression of YAP target genes.^6^ By contrast, non-phosphorylated and therefore active YAP enters the nucleus and binds to transcriptional factors.^7^ For years, factors controlling the Hippo pathway upstream of LATS kinase were elusive. However, recently several groups independently discovered that G protein-coupled receptors (GPCRs) on the surface of eukaryotic cells are able to act as modulators of Hippo signaling.^8, 9, 10^

One of the most pharmacologically relevant GPCRs is the Angiotensin II type 1 receptor (AT1R), which is linked to the G*α*_q/11_ subclass and is part of the renin–angiotensin–aldosterone system^11^ that is crucial for blood pressure and water homeostasis. The ligand Angiotensin II (Ang II) binds to the AT1R with high affinity and initiates different intracellular signaling pathways. The effect of Ang II can be blocked by AT1R blockers or by angiotensin-converting enzyme inhibitors.^12^ Angiotensin-converting enzyme inhibitors and AT1R blockers are clinically used for the treatment of hypertension and heart failure in patients. Interestingly, both blockers also show a protective effect on kidney function, especially in proteinuric patients.^13^ Podocytes as essential cells of the renal filtration barrier exhibit an endogenous renin–angiotensin–aldosterone system. As increasing evidence suggests that proteinuria is predominantly a disease of podocytes, it is assumed that the observed reno-protective effects could be – at least partially – due to a blockade of this podocyte-specific renin–angiotensin–aldosterone system.^14^

Here we analyzed the impact of the AT1R as an upstream regulator on the Hippo pathway. Ang II inactivates the Hippo pathway in HEK293T cells owing to an impaired LATS activity and a subsequent enhanced YAP nuclear shuttling. Surprisingly, in podocytes the Hippo pathway is constantly inactive and AT1R insensitive. However a reactivation of the Hippo pathway in these cells results in an increased level of apoptosis.

## Results

### The Angiotensin II receptor serves as an upstream regulator of the Hippo pathway

**Figure 1:**
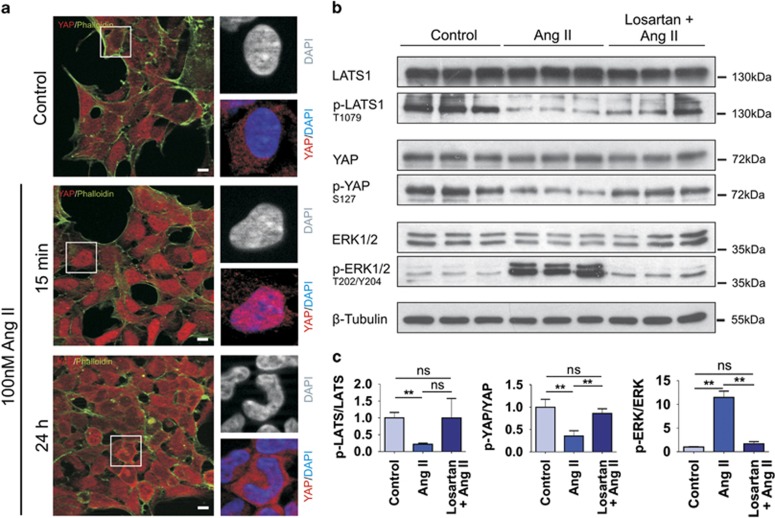
The Angiotensin II receptor AT1R is an upstream regulator of the Hippo pathway. (**a**) Immunofluorescence staining showed homogenous cytoplasmic as well as nuclear localization of YAP (red) in unstimulated AT1R-overexpressing HEK293 cells; DAPI (blue) marks the nuclei and Alexa 488 Phalloidin (green) the actin cytoskeleton. Ang II stimulation (100 nM) for 15 min led to translocation of YAP to the nuclei. This effect was not seen in cells after prolonged Ang II treatment (100 nM, 24 h). Scale bars represent 10 *μ*m. (**b**) Western blot analysis of sets of three independent extracts from AT1R-overexpressing HEK293 cells; unstimulated (left panel), stimulated (100 nM Ang II, 30 min, middle panel) or pretreated with the AT1R inhibitor Losartan (1 *μ*M for 4 h) prior to stimulation with Ang II (right panel). Immunological detection revealed an Ang II-dependent strong increase of ERK phosphorylation, accompanied by the dephosphorylation of LATS1 on T1079 and its downstream target YAP on S127. The specific inhibition of Ang II signaling by the AT1R blocker Losartan demonstrated that phosphorylation (ERK) and dephosphorylation effects (LATS/YAP) are solely due to Ang II stimulation. (**c**) The ratio between phosphorylated and total amount of the protein is calculated and indicated (mean and S.D., *t*-test, **P<0.01; ns: not significant)

AT1R belongs to the group of GPCRs that act as upstream receptors of Hippo signaling. To analyze a putative influence of the AT1R on Hippo signaling, we used previously established AT1R-overexpressing HEK293 cells (kindly provided by Dr R Lefkowitz^15^). In an immunofluorescence experiment, the YAP distribution in these cells was analyzed after the Ang II treatment using a YAP-specific antibody. Stimulation with 100 nM Ang II for 15 min resulted in a strong nuclear accumulation of YAP, which is lost after a prolonged stimulation of 24 h (Figure 1a). Quantitative western blot analysis from cells stimulated with 100 nM Ang II for 30 min using phospho-specific antibodies revealed a decrease of LATS phosphorylation at Threonine 1079 (T1079) and of YAP at Serine 127 (S127) compared with the control without Ang II treatment (Figures 1b and c). This effect could be almost completely blocked by simultaneous treatment with the AT1R-specific inhibitor Losartan, proving that this effect is AT1R-dependent. Stimulation of the HEK293 cells with Ang II also showed an increased ERK phosphorylation, which is a well-known target of Ang II signaling.^16, 17^ The dephosphorylation of YAP was time-dependent and was not detectable after a stimulation of 24 h (Supplementary Figure 1). Therefore, Ang II stimulation inhibits Hippo signaling in HEK293 cells by dephosphorylation, and thereby inactivation of LATS kinase, resulting in a consecutive dephosphorylation and nuclear accumulation of YAP.

### Inactivation of Hippo signaling is actin-dependent

**Figure 2:**
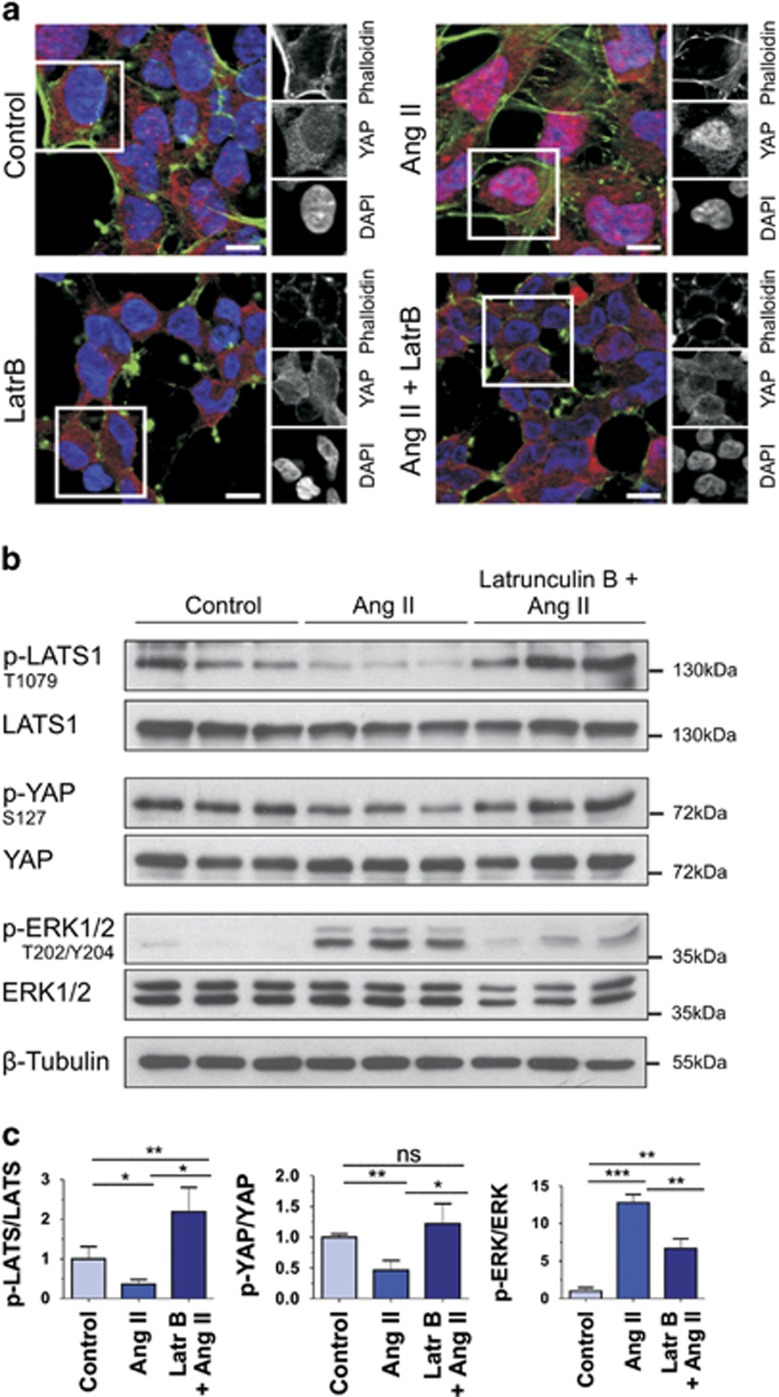
Inactivation of Hippo signaling by Ang II is actin-dependent. (**a**) HEK293 cells overexpressing AT1R were pretreated with Latrunculin B (500 nM) for 10 min prior Ang II stimulation (100 nM, 30 min). Immunofluorescence staining of endogenous YAP (red) showed that disruption of the actin cytoskeleton (green) by Latrunculin B blocked the Ang II-induced nuclear translocation of YAP; DAPI (blue) was used to visualize nuclei. Scale bars represent 10 *μ*m. (**b**) Western blot analysis of sets of three independent lysates from AT1R-overexpressing HEK293 cells untreated, treated with Ang II (100 nM, 30 min) or pretreated for 10 min with Latrunculin B (500 nM) prior Ang II stimulation (100 nM, 30 min). The disruption of the actin cytoskeleton blocks dephosphorylation of LATS1-T1079 and YAP-S127 and is accompanied by a reduced phosphorylation state of ERK T202/Y204. (**c**) The ratio between phosphorylated and total amount of the protein is calculated and indicated (mean and S.D., *t*-test,*P<0.05, **P<0.01, ***P<0.001; ns: not significant)

Previous studies had shown that disruption of the actin cytoskeleton is able to inhibit a GPCR-dependent activation of Hippo signaling,^9, 10^ indicating that the GPCR-Hippo signaling axis requires an intact actin cytoskeleton. In order to test whether the signaling induced by the AT1R is also actin-dependent, we pretreated the AT1R-overexpressing cells with the actin depolymerizing drug Latrunculin B before Ang II stimulation. Immunofluorescence staining revealed that nuclear YAP accumulation after Ang II treatment was prevented by actin cytoskeleton disruption with Latrunculin B (Figure 2a). Quantitative western blot analysis of corresponding cell lysates showed reduced YAP and LATS phosphorylation levels after Ang II treatment, which cannot be observed in cells pretreated with Latrunculin B (Figures 2b and c). These results imply that a proper actin cytoskeleton is a basic prerequisite of the GPCR-Hippo signaling axis.

### The Hippo pathway is inactive in podocytes and Ang II insensitive

**Figure 3:**
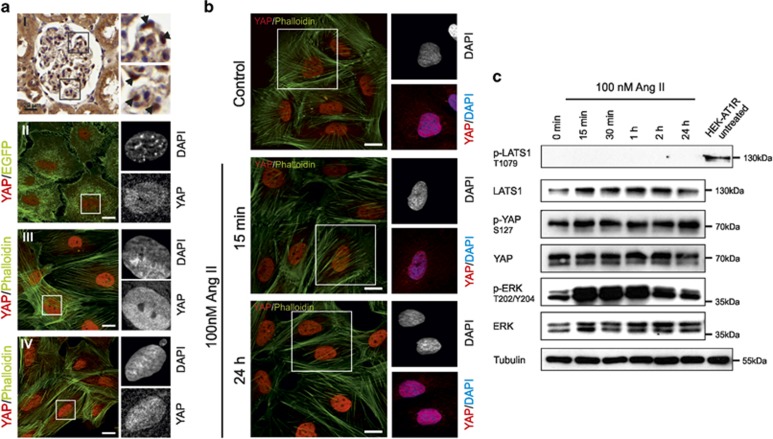
Hippo pathway is inactive in podocytes and Ang II insensitive. (**A**) *In vivo* YAP distribution in podocytes is mainly nuclear as well as in primary and in immortalized podocytes: (i) Immunohistochemical staining of YAP (brown) in a kidney section of a rat (arrows in small pictures indicates podocytes); Hematoxylin (blue) was used to visualize nuclei. (ii) Immunofluorescence staining of YAP (red) in isolated genetically marked primary mouse podocytes (EGFP, green). Immunofluorescence staining of YAP (red) in cells of an immortalized human podocyte cell line: differentiated human podocyte cell line (iii) and proliferating cells of the same podocyte cell line (iv). Phalloidin (green) was used to stain the actin cytoskeleton. (**B**) Immunofluorescence of AT1R-overexpressing podocytes stained for YAP localization (red) and actin (green). Podocytes were stimulated with 100 nM Ang II for 15 min or 24 h, non-stimulated cells served as control. (**C**) Western blot: cell extracts from AT1R-overexpressing podocytes were used to detect endogenous LATS1, p-T1079-LATS1, YAP, p-S127-YAP, ERK1/2, p-T202/Y204-ERK1/2 and *β*-tubulin, which served as loading control. Cells were treated with Ang II for the indicated time points. No change in the phospho-LATS and YAP signals was observed. HEK293 AT1R-overexpressing cells served as positive control for the p-T1079-LATS1 antibody. Phosphorylation of ERK1/2 (p-T202/Y204-ERK1/2) served as a positive control for the Ang II stimulation. Scale bars represent 20 *μ*m

As podocytes respond to Ang II *in vivo* ^18, 19^ and blockade of AT1R has reno-protective effects in patients, the influence of Ang II on the Hippo pathway was analyzed in further experiments in podocytes. Therefore, we first had to determine the status of the Hippo pathway in podocytes. For this, the *in vivo* YAP distribution was analyzed by immunohistochemistry staining of rat glomeruli (Figure 3a). In addition, we analyzed the YAP distribution in primary isolated mouse podocytes (Figures 3a and b) as well as in a human podocyte cell lines under proliferating and differentiated conditions (Figures 3c and d, Supplementary Figure 2). All approaches elucidated a predominant nuclear YAP staining of podocytes.

In contrast to glomerular podocytes *in vivo*, which respond to Ang II stimulation very sensitively,^18, 19^ the immortalized podocyte cell line^20^ displays a weaker reaction on Ang II stimulation *in vitro* because of partial loss of AT1R expression. In order to analyze the link between AT1R and Hippo signaling in podocytes in more detail, we created a human podocyte cell line, which stably overexpresses an N-terminally FLAG-tagged AT1R fusion protein. These cells displayed a robust (~100%) response to Ang II treatment compared with control human podocytes (~30%, Supplementary Figure 3). Stimulation with Ang II led to an increase of ERK phosphorylation and a significant increase of the intracellular calcium concentration, effects that could both be blocked by pre-incubation with the specific AT1R inhibitor Losartan. The detailed characterization of the stable podocyte cell line is given in Supplementary Figure 3.

Interestingly, Ang II treatment for 15 min or 24 h had no effect on YAP localization in human podocytes and in podocytes overexpressing AT1R (Figure 3b). Primary mouse podocytes displayed no change in YAP distribution over prolonged stimulation with Ang II (Supplementary Figure 2). Also a prolonged Ang II stimulation of podocytes overexpressing AT1R for 7 days did not alter the nuclear YAP distribution (Supplementary Figure 4). The nuclear accumulation of YAP indicates that the Hippo signaling pathway is turned off in podocytes, even being independent of prolonged Ang II stimulation. However, AT1R-overexpressing podocytes displayed an increased phosphorylation of ERK, showing a robust activation of the Ang II/AT1R pathway in podocytes after Ang II treatment (Figure 3c). In contrast to AT1R-overexpressing HEK293 cells, the p-YAP/YAP ratio remained unchanged in podocytes during Ang II stimulation. In addition, we observed nearly no phosphorylation of LATS1 at T1079 (Figure 3c), which is essential for LATS kinase activity,^21^ suggesting that predominant nuclear YAP distribution correlates with an inactivated LATS kinase in podocytes.

### Disruption of the actin cytoskeleton in podocytes results in a cytoplasmic redistribution of YAP

**Figure 4:**
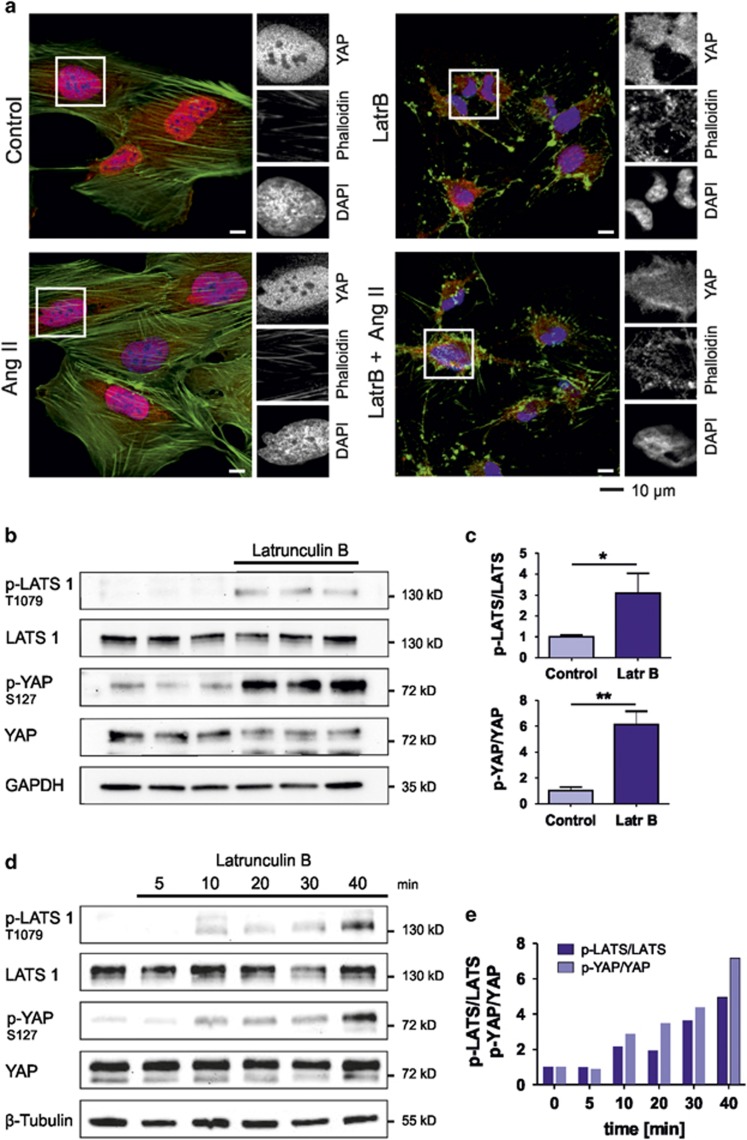
Disruption of actin cytoskeleton in podocytes results in a cytoplasmic redistribution of YAP. (**a**) The disruption of the actin cytoskeleton by pretreatment with Latrunculin B (500 nM) for 10 min led to a cytosolic translocation of endogenous YAP (red) in podocytes. This effect could not be inhibited or reversed by stimulation with Ang II; DAPI (blue) was used to visualize nuclei. Scale bars represent 10 *μ*m. (**b**) Cell extracts were used to detect endogenous LATS1, p-T1079-LATS1, YAP, p-S127-YAP. Western blot analysis of a set of three independent extracts from podocytes treated for 40 min with Latrunculin B (500 nM) revealed that disruption of the actin cytoskeleton led to an increase of the phosphorylation of LATS1-T1079 and YAP-S127. Endogenous GAPDH protein serves as loading control. (**c**) The ratio between phosphorylated and total amount of the protein is calculated and indicated (mean and S.D., *t*-test,*P<0.05, **P<0.01). (**d**) Cell extracts were used to detect endogenous LATS1, p-T1079-LATS1, YAP, p-S127-YAP and *β*-tubulin, which served as loading control. Western blot analysis of cell extracts from podocytes treated with Latrunculin B for indicated time points showed increasing phosphorylation of LATS1-T1079 and YAP-S127. (**e**) The ratio between phosphorylated and total amount of the protein is calculated and indicated

YAP localization and phosphorylation is influenced by the status of the actin cytoskeleton.^22, 23^ This is of special significance for podocytes, as the formation of the renal filtration barrier depends on highly branched actin-rich structures called foot processes.^24^ To investigate the influence of depolymerized actin on the YAP distribution, we treated the human podocytes with Latrunculin B (Figure 4a). Using immunofluorescence analysis, we observed that the depolymerization of actin results in a shift from nuclear YAP staining into a more cytoplasmic distribution (Figure 4a). As expected, Ang II was not able to influence the Latrunculin B-dependent nuclear export of YAP in podocytes. This change in YAP distribution after Latrunculin B treatment could also be observed in differentiated podocytes and in a mouse podocyte cell line (Supplementary Figures 5 and 6), indicating that this effect is not limited to one cell line. Western blot analysis of Latrunculin B treated cells revealed that the increased cytoplasmic distribution strongly correlates with an increased phosphorylation of LATS kinase at T1079 and its downstream target YAP at S127 (Figures 4b and c). The increase of LATS1 phosphorylation at T1079 starts 10 min after Latrunculin B treatment and increased over time (Figures 4 and e), indicating that the prolonged disruption of actin cytoskeleton stimulates Hippo signaling by activating LATS kinase.

### LATS-dependent reactivation of Hippo pathway in podocytes results in a nuclear export of YAP

**Figure 5:**
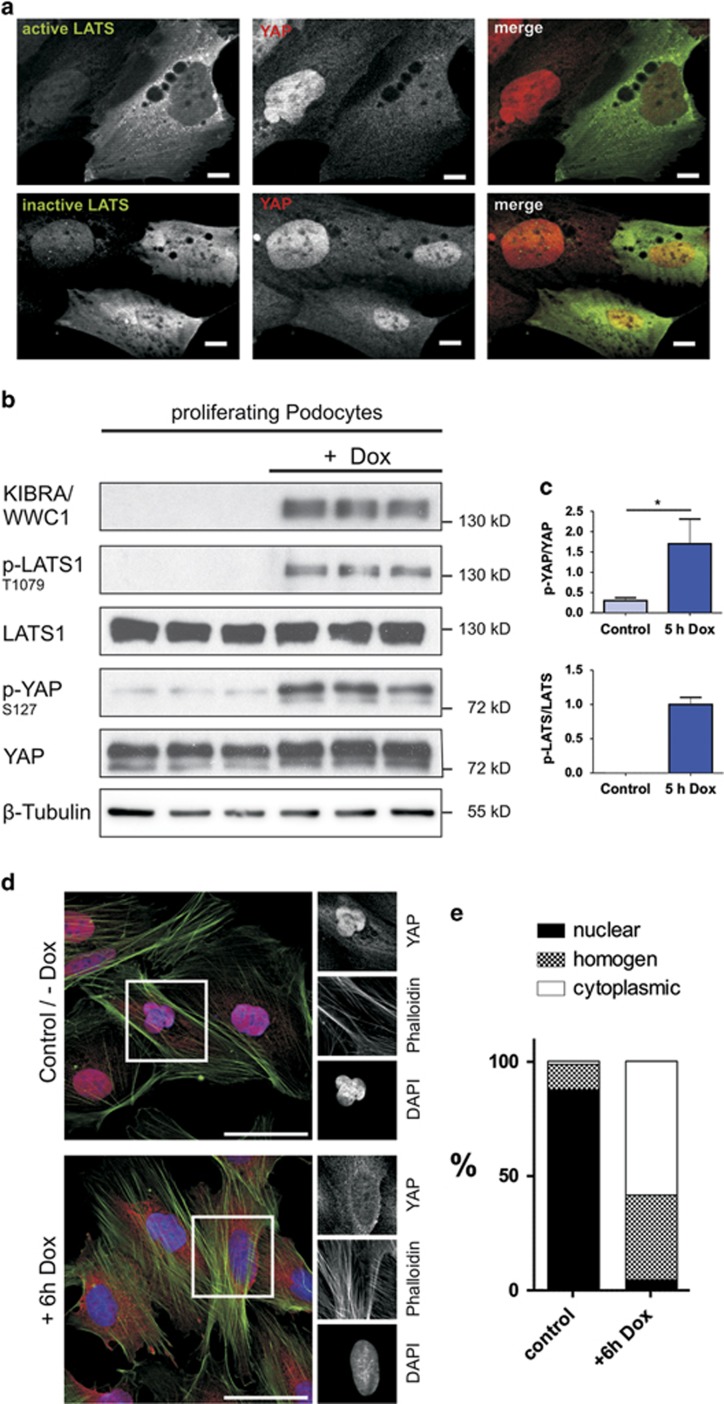
LATS-dependent reactivation of Hippo pathway in podocytes results in a nuclear export of YAP. (**a**) Transient expression of a permanent active LATS2 mutant (T1041E, green) and a permanent inactive LATS2 mutant (T1041A, green) each tagged with a FLAG epitope in podocytes led to an increase of cytoplasmic YAP (red) localization in cells expressing the permanent active mutant but not the permanent inactive mutant. Scale bars represent 10 *μ*m. (**b**) Indirect activation of LATS1 kinase due to inducible KIBRA/WWC1 overexpression (doxycycline treatment, 125 ng/ml for 5 h) was used to verify the link between LATS activation and nuclear export of YAP. Cell lysates of doxycycline-treated and -untreated cells were utilized to detect endogenous LATS1, p-1079-LATS1, YAP, p-S127-YAP, KIBRA/WWC1 and *β*-tubulin, which served as loading control. Overexpression of KIBRA/WWC1 in podocytes results in a robust increase in LATS and YAP phosphorylation. (**c**) The ratio between phosphorylated and total amount of the protein is calculated and indicated (mean and S.D., *t*-test,*P<0.05). (**d**) Immunofluorescence staining of YAP (red) in podocytes after induction of the KIBRA/WWC1 overexpression by doxycycline for 6 h: overexpression of KIBRA/WWC1 leads to a cytoplasmic localization of YAP; Phalloidin (green) marks the actin cytoskeleton, DAPI (blue) was used to visualize nuclei. Scale bars represent 50 *μ*m. (**e**) Quantitative evaluation of 100 cells shown in **c** proves the general translocation of YAP. For each cell, the localization of YAP was estimated as nuclear, cytoplasmic or homogeneous (comparable levels of nuclear and cytoplasmic staining)

In order to confirm that the predominant nuclear YAP distribution is a result of an inactive LATS kinase and to show that active LATS is able to promote a nuclear export of YAP into the cytosol of podocytes, we transiently transfected podocytes with an expression plasmid, encoding a permanently active and a permanently inactive LATS kinase (LATS2 T1041E and LATS2 T1041 A). LATS1 and LATS2 share a high homologous sequence and can both phosphorylate YAP at S127.^5, 25^ The phosphorylation site T1079 from LATS1 correlates with T1041 in LATS2. Immunofluorescence analysis showed that cells expressing the active mutant of LATS display a cytoplasmatic YAP distribution, whereas inactive LATS did not influence the YAP localization (Figure 5a).

We recently elucidated that an induced expression of KIBRA/WWC1 in HEK293T cells results in highly increased levels of phosphorylated LATS at T1079, which is similar to a strong activation of endogenous LATS kinase activity in these cells.^26^ Therefore, we established a human podocyte cell line inducibly overexpressing KIBRA/WWC1 to prove that an activation of LATS kinase by a known activator triggers YAP phosphorylation and nuclear export. As expected, reactivation of the Hippo pathway by overexpression of KIBRA/WWC1 significantly increased phosphorylation of LATS and YAP in podocytes comparable with the published data in HEK293T^26^ (Figures 5b and c and Supplementary Figure 7a). In addition, immunofluorescence studies in podocytes showed increasing amounts of cytoplasmic (inactive) YAP as a result of KIBRA/WWC1 expression (Figures 5d and e) comparable to KIBRA/WWC1 overexpression in HEK293T cells (Supplementary Figures 7c and d).

Proliferating podocytes *in vitro* share features of proliferating/immature podocytes *in vivo*.^27^ The human podocyte cell line is able to differentiate after a temperature shift to 37 °C for 10 days, leading to a stop of proliferation, a redistribution of the cytoskeleton and an increased expression of podocyte marker proteins^20^ (Supplementary Figure 7a). The phosphorylation of LATS could be further enhanced by KIBRA expression in both proliferating and differentiated podocytes, which led in both conditions to an enhanced phosphorylation of the LATS target YAP. Interestingly, in these differentiated podocytes, the total LATS expression is reduced in comparison with proliferating podocytes, whereas the total YAP expression is increased (Supplementary Figures 7a and b). Although the total YAP level increased in differentiated podocytes, the p-YAP/YAP ratio was unchanged between proliferating and differentiated podocytes. We could also observe a slight increase of phosphorylation at T1079-Lats in differentiated podocytes compared with proliferating podocytes, which only showed a weak phospho-signal at T1079 (Supplementary Figure 7a). As shown before, differentiated podocytes showed an enhanced KIBRA/WWC1 expression in comparison with proliferating podocytes (under control conditions without doxycycline treatment) (Supplementary Figures 7a and b).^28^ As expected, the level of Podocin, a podocyte marker protein, increased in differentiated podocytes compared with proliferating podocytes. During activation of Hippo signaling, the expression level of Podocin was not changed after 6 h or 24 h of KIBRA/WWC1 expression (Supplementary Figure 7a). In addition, we could detect the expression of the YAP paralog TAZ with the alternative name transcriptional coactivator with PDZ-binding motif (WWTR1) in our human podocyte cell line (Supplementary Figure 7a).

### Overexpression of the Hippo pathway component KIBRA/WWC1 leads to an increased level of apoptosis in podocytes but not in HEK293T cells

**Figure 6:**
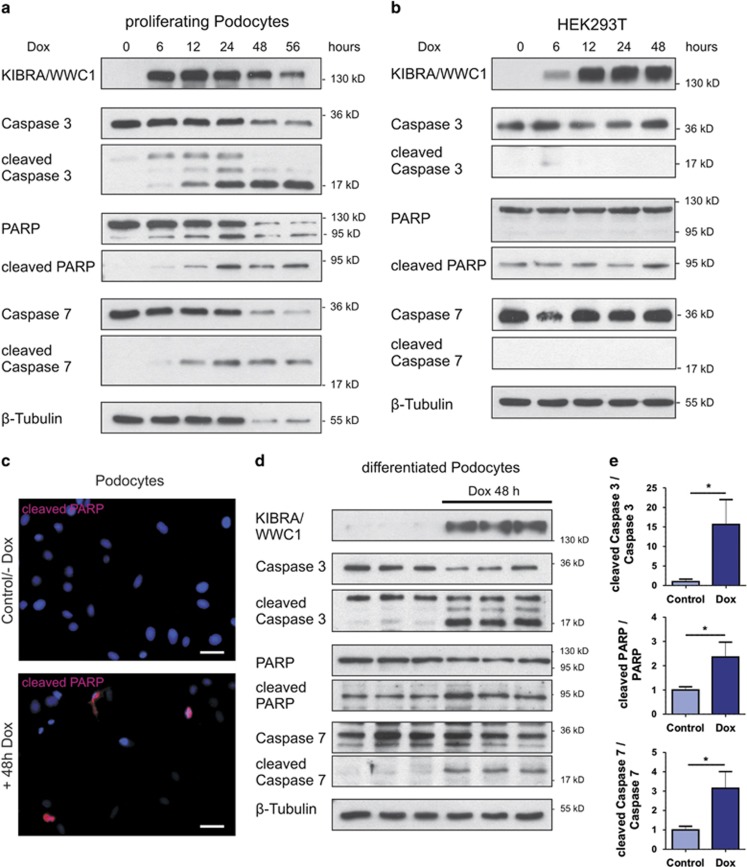
Overexpression of the Hippo pathway component KIBRA/WWC1 leads to an increased level of apoptosis in podocytes but not in HEK293T cells. (**a** and **b**) Induced KIBRA/WWC1 expression in podocytes (**a**), but not in HEK293T cells (**b**) leads to increased apoptosis levels. Lysates of cells induced by treatment with doxycycline (125 ng/ml) for the indicated times were used to detect endogenous Caspase 3, Caspase 7, PARP, and their cleaved versions cleaved Caspase 3 and 7, and cleaved PARP, which are well-established markers for apoptosis. *β*-tubulin served as loading control. Induced KIBRA/WWC1 overexpression caused the increase in levels of apoptosis markers cleaved Caspase 3, cleaved Caspase 7 and cleaved PARP, accompanied by the stepwise decrease of the non-cleaved version of these proteins. The decreased *β*-tubulin signal illustrates the loss of the total amount of podocytes in which the Hippo signaling was reactivated (via KIBRA/WWC1 overexpression) for 48 h or 56 h. By contrast in HEK293T cells, the expression of the apoptotic marker and *β*-tubulin was stable for the entire measurement. (**c**) Immunofluorescence of podocytes analyzed in **a** show a nuclear cleaved PARP (red) staining after induction of KIBRA/WWC1 expression (nuclear staining: DAPI). Scale bars represent 10 *μ*m. (**d**) The reactivation of Hippo signaling via induced KIBRA/WWC1 expression could also be confirmed in differentiated podocytes and again led to an accumulation of apoptotic marker proteins. (**e**) The statistic evaluation of the apoptotic marker proteins. (mean and S.D., *t*-test, *P<0.05)

As the Hippo pathway controls the balance between proliferation and apoptosis, we examined the effect of an increased Hippo signaling due to an induced overexpression of the upstream regulator KIBRA/WWC1 in podocytes. Interestingly, we observed a loss of podocytes starting 24 h after induction of KIBRA/WWC1 overexpression and speculated that the observed podocyte depletion might be due to an increased level of apoptosis. Indeed, using western blot analysis with cellular extracts from these cells, we detected increasing signals for apoptotic marker proteins cleaved PARP, cleaved Caspase 3 and cleaved Caspase 7 in podocytes in contrast to HEK293T cells, where the levels of these markers remained unchanged after induction of KIBRA/WWC1 overexpression (Figure 6a and b, see also Supplementary Figure 8). In addition, immunofluorescence analysis showed positive signals for nuclear cleaved PARP only in those podocytes, in which the Hippo pathway was reactivated by the induction of KIBRA/WWC1 overexpression (Figure 6c +Dox) and not in control cells (Figure 6c −Dox). The increased susceptibility of podocytes to apoptosis upon reactivation of the Hippo pathway is independent from the differentiation status of these cells, as also differentiated podocytes showed a loss of cells after 24 h of KIBRA/WWC1 overexpression and positive signals for apoptosis markers (Figures 6d and e). Thus reactivation of the Hippo pathway by activating LATS kinase is accompanied by elevated apoptosis levels in proliferating as well as in differentiated podocytes.

## Discussion

This study shows for the first time that Ang II binding to the AT1R is capable to inhibit Hippo signaling and to activate YAP. As GPCR's coupling to the G protein subclass G*α*_q/11,_ in general, are able to activate YAP,^10, 29^ we therefore expected the same influence of the AT1R, which is mainly coupling to G*α*_q/11_. Stimulation of the AT1R with Ang II showed a decreased phosphorylation of LATS, which was accompanied by a decreased phosphorylation of its target YAP. These signaling events were actin-dependent, which could be shown using Latrunculin B as actin destabilizing substance. The disruption of F-actin inhibited the dephosphorylation of both, YAP and LATS kinase. The specific AT1R inhibitor Losartan was able to inhibit the Ang II-induced signaling of the Hippo pathway and also of ERK activation, which was used as a known downstream effect of Ang II stimulation.

Interestingly, the comparison between AT1R-overexpressing HEK293 cells and podocytes revealed a podocyte-specific regulation of Hippo signaling, as Ang II treatment in HEK293 cells triggers the shift from cytoplasmic into nuclear YAP distribution. In contrast, YAP localization in podocytes remains predominantly nuclear, independent from AT1R activation via Ang II treatment. We observed this predominant nuclear YAP distribution *in vivo* and *in vitro*. Our data suggest that the nuclear YAP localization originates most probably from the inactive LATS kinase represented by a lack of a sufficient LATS phosphorylation of the crucial T1079 residue. This hypothesis was strengthen by our findings that either ectopic expression of permanent active LATS kinase mutant or overexpressing of the positive LATS regulator KIBRA/WWC1 are sufficient to trigger YAP phosphorylation and cytoplasmatic retention.

Intriguingly, a reactivation of the Hippo signaling mediated through enhanced LATS kinase activity can also be induced by Latrunculin B treatment, indicating that in podocytes, actin depolymerization is a crucial regulator of the Hippo pathway upstream of LATS kinase. It is currently under debate whether or not actin polymerization controls Hippo signaling in a LATS-dependent or -independent manner.^22, 30^ Thus, our data suggest, at least in podocytes, the existence of kinases, which specifically activate LATS by phosphorylation of T1079 after disruption of the actin cytoskeleton. Alternatively, LATS-specific phosphatases keeping LATS in the non-phosphorylated form could be inactivated during actin-remodeling processes. This is interesting, as in podocytes, the physiological function is highly connected to actin-dependent processes.^24^ A novel link between LATS kinase and actin dynamics was discussed from publications, describing the interaction of LATS with Lim-domain-kinase (LIMK) and the family of angiomotin proteins.^31^ LIMK is a cytoskeletal protein kinase, which organizes actin dynamics by phosphorylating and inactivating Cofilin. LATS kinase phosphorylates and inactivates LIMK, raising the possibility that LATS by itself can influence actin dynamics.^32^ Cofilin is also associated with the regulation of Hippo signaling, as it was found in a siRNA screen for actin influencing-proteins with a Hippo pathway-specific readout.^33^ Overexpression or silencing of Cofilin expression in cells modifies Hippo signaling by influencing actin dynamics and is able to overcome cell confluence-dependent Hippo pathway activation, highlighting the importance of actin cytoskeleton. In this context, it is interesting that proteins-like LIMK, Cofilin or angiomotin can possibly be the unknown link between actin dynamics and LATS kinase regulation in podocytes. In summary, actin dynamic takes a central position in Hippo signaling because it not only transfers the signal from GPCR's, but also connects mechanical forces to Hippo signaling. In general, these forces can result from the sjpegness of the extracellular matrix, from the geometry of the cell and from forces created from cell density.^23^

Recently, Campbell *et al.*^34^ have postulated that in postmitotic podocytes, nuclear YAP functions predominantly as pro-survival factor. Our data support this hypothesis and emphasize the role of Hippo signaling in podocytes, as a reactivation of the Hippo pathway by overexpressing KIBRA/WWC1 causes increased apoptosis in proliferating and differentiated podocytes. In addition, the core components of the pathway are differentially expressed in proliferating and differentiated podocytes, as LATS expression is reduced and YAP expression is increased in differentiated podocytes. The pYAP/YAP ratio was not changed during the differentiation. These facts indicate that even in postmitotic podocytes, the inactivated Hippo pathway has an important role for podocyte homeostasis. *In vivo* at the renal filtration barrier apoptotic podocytes cannot be replaced because of their postmitotic status. A continuous loss of podocytes due to apoptosis leads to glomerulosclerosis, a decrease of kidney function and finally to the dependence of dialysis.^35^ Thus, the characterization of new signaling pathways, influencing podocyte function or gaining deeper insights into their survival strategies, are of high interest, as podocyte-specific therapy to treat podocytopathies are insufficient, yet.

The Hippo pathway is connected to renal disease and development. Happé *et al.*^36^ described the YAP distribution throughout the kidney, with focus on tubular structures. They found a variable localization of YAP with a nuclear YAP staining in distal tubules and collecting duct, but not in proximal tubules. Cysts derived from kidney tumor tissue or from cystic kidney disease show a nuclear YAP distribution. Interestingly, the kidney-specific YAP and TAZ knockout in an early developmental stage of the kidney on the other hand leads to alterations with cyst generation.^37^ Also proteins that cause nephronophtisis, a cystic degenerative kidney disease, are able to influence Hippo signaling.^38, 39^ Altogether these data indicate connections between changes in Hippo pathway activity and different kidney diseases.

**Figure 7:**
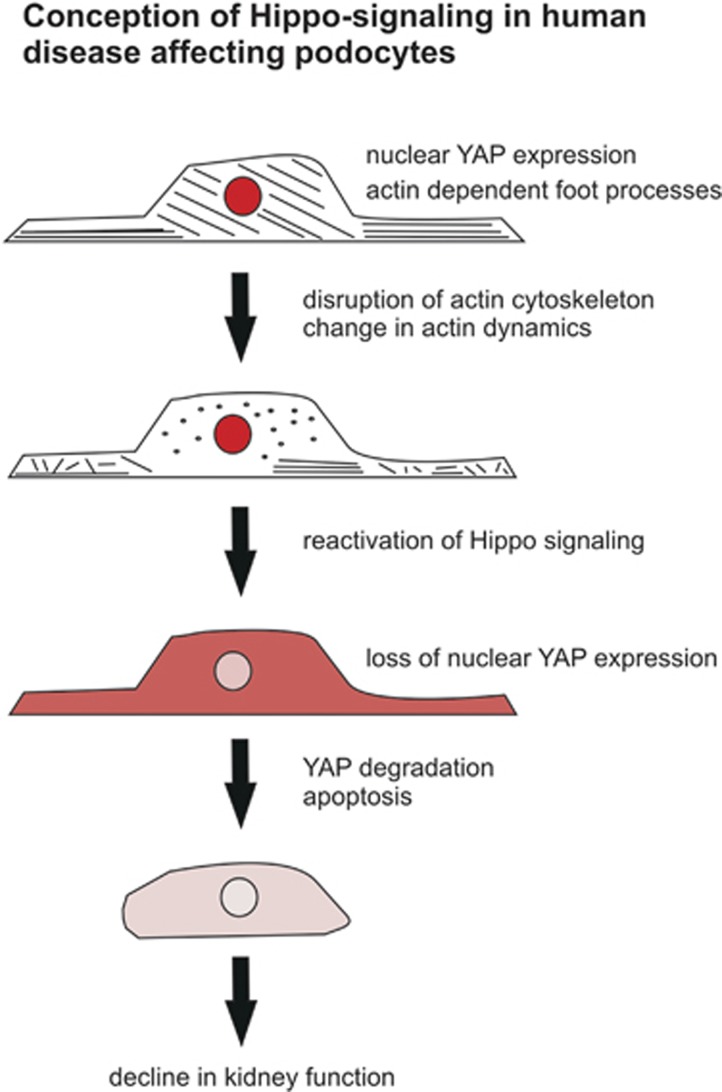
Proposed model of the regulation and effects of hippo pathway in podocytes. Podocytes display a highly organized cytoskeleton, which is important for the complex structure of the cells. Actin dynamics or the disruption of the actin cytoskeleton impairs the complex structure of these cells, leading to a loss of physiological functions. The disruption of the actin cytoskeleton activates Hippo signaling by activating LATS kinase, which changes the predominant nuclear YAP signal to a more cytoplasmic distribution. Reactivation of Hippo signaling by activating LATS kinase is sufficient to induce apoptosis in podocytes, but not in HEK293T cells

Podocytes from proteinuric patients frequently show a loss of interdigitated foot processes accompanied by a disruption of the actin cytoskeleton. Our data therefore support a hypothesis that a change in actin dynamics under pathological conditions leads to an activation of LATS (T1079 phosphorylation) and to a subsequent cytoplasmic YAP distribution that, in turn, triggers podocyte apoptosis (see Figure 7). Future studies will show whether our findings in different cell culture systems will come true *in vivo*, especially in the context of Ang II signaling and its discussed contribution to podocyte apoptosis.^14, 40, 41^ However, our data indicate that LATS kinase might be an essential signaling hub between actin dynamics and Hippo signaling in podocytes. Hence, the role of Hippo signaling in postmitotic cells will be an important issue to discover as it can be an interesting target for pharmacological intervention in podocytes in the future.

## Materials and Methods

### Constructs and cloning

We used the previously described human podocyte cDNA library to amplify an expression cassette, which encodes for an N-terminal 3 × FLAG-tagged human AT1R, and cloned it into the retroviral expression vector pQCXIP to establish a stable AT1R-overexpressing human podocyte cell line.^28, 42^ The primers used were 3 × FLAG-AT1R forward (5′-caccggatccatggactacaaggaccacgacggcgattacaaggaccacgacatcgactacaaggacgacgatgacaagattctcaactcttctactgaagatgg-3′) and AT1R reverse (5′-gaattctcactcaacctcaaaacatgg-3′), respectively.

The cloning of the construct for WWC1/KIBRA inducible cell lines has been described before.^26^ The construct for transient expression of the permanent LATS2 kinase FLAG-LATS2 T1041E K was a kind gift from Dr McCollum.^43^ All constructs were verified by sequencing.

### Cell culture

Human immortalized podocytes (AB8/13) (kindly provided by M Saleem), HEK293T (available at Thermo Scientific, Waltham, MA, USA) and Retro-X packing cell line GP2-293 (available at Clontech, Saint-Germain-en-Laye, France) were cultivated as described earlier.^20, 28, 42^ In brief, AB8/13 cells were grown in standard RPMI 1640 medium containing 10% fetal calf serum and supplements either at the permissive temperature of 33 °C (in 5% CO2) to promote cell propagation or at the non-permissive temperature of 37 °C (in 5% CO2) to allow the terminal differentiation. Retro-X packing cell line GP2-293 (Clontech) and HEK293T cells were cultivated in standard medium (Dulbecco's modified Eagle medium supplemented with 10% fetal calf serum and 1% antibiotics (Pen/Strep).^28^ For transient transfection, HEK293T or Retro-X packing cell line GP2-293 (Clontech) were transfected by the calcium phosphate method as described earlier.^28^ Podocytes were transiently transfected using Lipofectamine 2000 (Invitrogen, Thermo Fisher Scientific Inc., Waltham, MA, USA) according to the manufacturer's instructions. For activation of the AT1R, Angiotensin II (Sigma, Munich, Germany) was solved in water and added to the cells for the indicated times at a final concentration of 10–100 nM.

### Generation of stable cell lines

The generation of stable cells using retroviral (Retro-X, Clontech) or lentiviral systems (pINDUCER21) have been described in more detail earlier.^42^ For stable retroviral transduction, GP2-293 cells (10 cm dish) were simultaneously transfected with 5 *μ*g pVSV-G envelope plasmid (Clontech) and 5 *μ*g pQCXIP-3xFLAG AT1R expression plasmids to obtain recombinant virus. In case of generating stable doxycycline-inducible cell lines, we used the recently established pINDUCER system with a modified pINDUCER21 plasmid.^26, 44^ In this case, a 10-cm dish with 50% confluent HEK293T cells was simultaneously transfected with 10 *μ*g of the pINDUCER21 Puro vector with a KIBRA/WWC1 insert and with 6.5 *μ*g psPAX2 (addgene plasmid #12260) and 3.5 *μ*g pMD2.G (addgene plasmid # 12259). In both cases (retro- as well as lentiviral-based transductions), the medium of transiently transfected GP2-293 or HEK293T cells was changed after 6–8 h and cells were grown for additional 72. After that time, the virus-containing supernatant was collected and filtered through a sterile 0.45 *μ*m syringe driven filter unit (Millipore, Schwalbach am Taunus, Germany). Subsequently, HEK293T cells or podocytes (target cells, growing in 6-well dishes) were infected for 24 h using one volume (up to 2 ml) of fresh Dulbecco's modified Eagle medium medium and one volume of the virus-containing filtrate supplemented with polybrene (final concentration 8 *μ*g/ml). Thereafter, the virus-containing medium was replaced by fresh medium and cells were regenerated for 24 h. This transduction procedure was repeated once. Cells were selected after a second regeneration period by puromycin (4 *μ*g/ml for HEK293T and 2 *μ*g/ml for podocytes). In pINDUCER21-based cell lines protein expression was induced by adding 125 ng/ml doxycycline to the medium. The overexpression of target proteins was verified by western blot analysis.

### Preparation of cell lysates and western blot analysis

Quantitative western blot analysis was done as described before.^28^ In brief, for the quantitative western blot assays, cells were grown on dishes and then scraped into 1x Laemmli (4% SDS, 5% 2-mercaptoethanol, 10% glycerol, 0.002% bromophenol blue, 0.0625 M Tris-HCl; pH 6.8). After boiling for 5 min, the cells were pushed through a 20-gauge needle, and equal volumes of cell lysates were separated on 8–15% SDS-PAGE gels (Biorad, Munich, Germany). Proteins were transferred to a PVDF membrane (Millipore) and incubated for 1 h at room temperature in blocking buffer (5% BSA powder dissolved in TBS containing 0.05% Tween-20 (TBS-T)). The lysates were equalized using *β*-tubulin (Sigma) or GAPDH (Covance, Princeton, NJ, USA) as loading controls. The monoclonal mouse antibody directed against YAP was from Santa Cruz (Heidelberg, Germany, sc-101199). Antibodies against LATS1 (#3477), LATS2 (#5888), p-LATS1-T1079 (#8654) p-MST1 (#3682), p-MST1/2-T183/180 (#3681), p-YAP-S127 (#4911), TAZ (#2149) and antibodies against ERK1/2 (#4695) and p-ERK1/2-T202/Y204) (#D13.14.4E) were purchased from Cell Signaling (Frankfurt, Germany). The antibody against Podocin was from Sigma (P0372). Fluorochrome-conjugated secondary antibodies coupled to the fluorescent dyes Alexa 488 and 594 were purchased from Invitrogen. Horseradish peroxidase-conjugated secondary antibodies were purchased from Dianova (Hamburg, Germany). All primary antibodies were used in a 1 : 1000 dilution in TBS-T and incubated at 4 °C overnight or for 1 h at room temperature. After washing three times with TBS-T, the membrane was incubated with horseradish peroxidase-coupled secondary antibodies (Jackson Immunoresearch, Westgrove, PA, USA) diluted 1 : 2000 in 5% BSA powder dissolved in TBS-T for 30 min at room temperature. Afterwards, the blot was washed three times with TBS-T. Chemiluminescence detection reagent (Roche, Mannheim, Germany) was used to develop the western blot.

### Quantification of western blot signals

Signals derived from the same immunoblot were densitometrically quantified using ImageJ (http://rsbweb.nih.gov/ij/). Signals derived from phospho-specific antibodies were analyzed in relation to the signals derived from the complete amount of protein. The evaluation was done using GraphPad prism (GraphPad software). All data show the S.D. of at least three independent experiments and were analyzed using unpaired students *t-*test: *P<0.05; **P<0.01; ***P<0.001.

### Apoptosis measurements

Apoptosis was monitored by quantitative western blot analysis, as outlined above, using the antibodies against full-length and the corresponding apoptosis-associated cleaved versions. The antibodies against PARP (#9542), cleaved PARP (#9544), Caspase 3 (#9665) cleaved Caspase 3 (#9664), Caspase 7 (#9492) and cleaved Caspase 7 (#8438) were from Cell signaling. To induce apoptosis in AT1R-overexpressing podocytes, we used 1 *μ*M Staurosporine (Sigma) over a time period of 3 and 24 h.

### Immunofluorescence analysis

AB8/13 podocytes and HEK293T cells were cultured on cover slips for indirect immunofluorescence analysis. For actin depolymerization experiments, Latrunculin B (Calbiochem, San Diego, CA, USA) was added for the indicated times in a concentration of 0.1–1 *μ*M. Cells were fixed with 4% paraformaldehyde supplemented with 4% sucrose in PBS for 20 min. All steps were performed at room temperature. Samples were washed with PBS and incubated with 50 mM NH_4_Cl in PBS to quench reactive amino groups. After continued washing, cover slips were permeabilized with PBS containing 0.2% gelatin and 0.2% TritonX-100 (PBS-TG). Then, samples were blocked with 10% goat serum diluted in PBS-TG for 20 min. Immunofluorescence staining was performed by incubating the cover slips for one hour with primary antibodies against YAP (Santa Cruz, 63.7, sc-101199) or cleaved PARP (#9544), diluted in PBS-TG containing 2% goat serum. Afterwards, cover slips were washed in PBS-TG and incubated with fluorochrome-conjugated secondary antibodies diluted 1 : 1000 (Alexa Fluor 595, Molecular Probes, Eugene, OR, USA). For visualizing polymerized actin the second antibody dilution also contained Alexa Fluor 488 Phalloidin 1 : 100 (Molecular Probes). Nuclei of cells were stained with DAPI (dilution 1 : 5000). After washing with PBS, cover slips were rinsed in distilled water and cells were mounted in Mowiol. Samples were examined with an Axio Observer Z1 microscope and ApoTome technology (Zeiss, Oberkochen, Germany; objective: EC Plan Neofluar 40x /1.30*Oil DIC M27) using Axio Vision 4.7.

### Immunhistochemistry analysis

After antigen retrieval with 0.01 M citric acid buffer (pH 6,0, boiled for 3 min) paraffin-embedded kidney sections (5 *μ*m) were incubated with a rabbit polyclonal antibody (1 : 100 in 1% BSA) against Yap (Santa Cruz, H125, sc-15407) overnight. This was followed by incubation with a biotinylated secondary antibody against rabbit (1 : 200, Vector Laboratories, Burlingame, CA, USA) in PBS. Finally, the sections were stained by incubation with avidin-biotin peroxidase (Vector Laboratories) and reaction with DAB. Nuclei were stained with hematoxilin.

### Isolation of murine primary podocytes

Genetic labeling of murine podocytes was performed as described earlier.^45^ In brief, the mTomato/mEGFP reporter gene^46^ mouse was bred with *NPHS2-Cre* mouse^47^ to induce a Cre-recombinase-dependent florescence switching from red (mTomato) to green (mEGFP) fluorescence in podocytes. EGFP-labeled podocytes were isolated and further analyzed by immunofluorescence for YAP localization.

The kidneys of 8–14-week-old mice were removed and then decapsulated. The cortex was isolated and minced as described previously.^48^ The minced tissue was gently pushed through a sieve of 100-*μ*m mesh and then pipetted through a 80-*μ*m sieve. The filtrate was then pipetted onto a 40-*μ*m sieve. The glomeruli held back on this sieve were rinsed off the filter and resuspended in RPMI culture medium with 10% FBS. For immunoflourescence, they were cultured on cover glass chips coated with collagen IV for ~7 days until podocytes started to grow out of the glomerula.

## Abbreviations

GPCRs: G protein-coupled receptors
LATS: large tumor suppressor kinase
YAP: Yes-associated protein
AT1R: angiotensin II type 1 receptor
Ang II: angiotensin II
HEK cells: human embryonic kidney cells
Yki: yorkie
ERK: extracellular-signal regulated kinase
RAAS: renin–angiotensin–aldosterone–system
ARB: angiotensin II typ 1 receptor blockers
ACEi: angiotensin-converting enzyme inhibitors
TAZ: transcriptional coactivator with PDZ-binding motif
KIBRA: kidney and brain protein
WWC1: WW domain-containing protein 1
LIMK: Lim-domain-kinase
AMOT: angiomotin
DAPI: 4′,6-Diamidin-2-phenylindol
EGFP: enhanced green fluorescent protein
GAPDH: glyceraldehyde-3-phosphate dehydrogenase
SD: standard deviation
PARP: poly ADP ribose polymerase
CoIP: Coimmunoprecipitation
FCS: fetal calf serum
Latr B: Latrunculin B
Dox: doxycycline
