# Supplementary figures and images for: The Hippo pathway is controlled by Angiotensin II signaling and its reactivation induces apoptosis in podocytes
Source: Cell Death Dis. 2014 Nov 13;5(11):e1519–. doi: 10.1038/cddis.2014.476 (PMC4260734; doi:10.1038/cddis.2014.476)

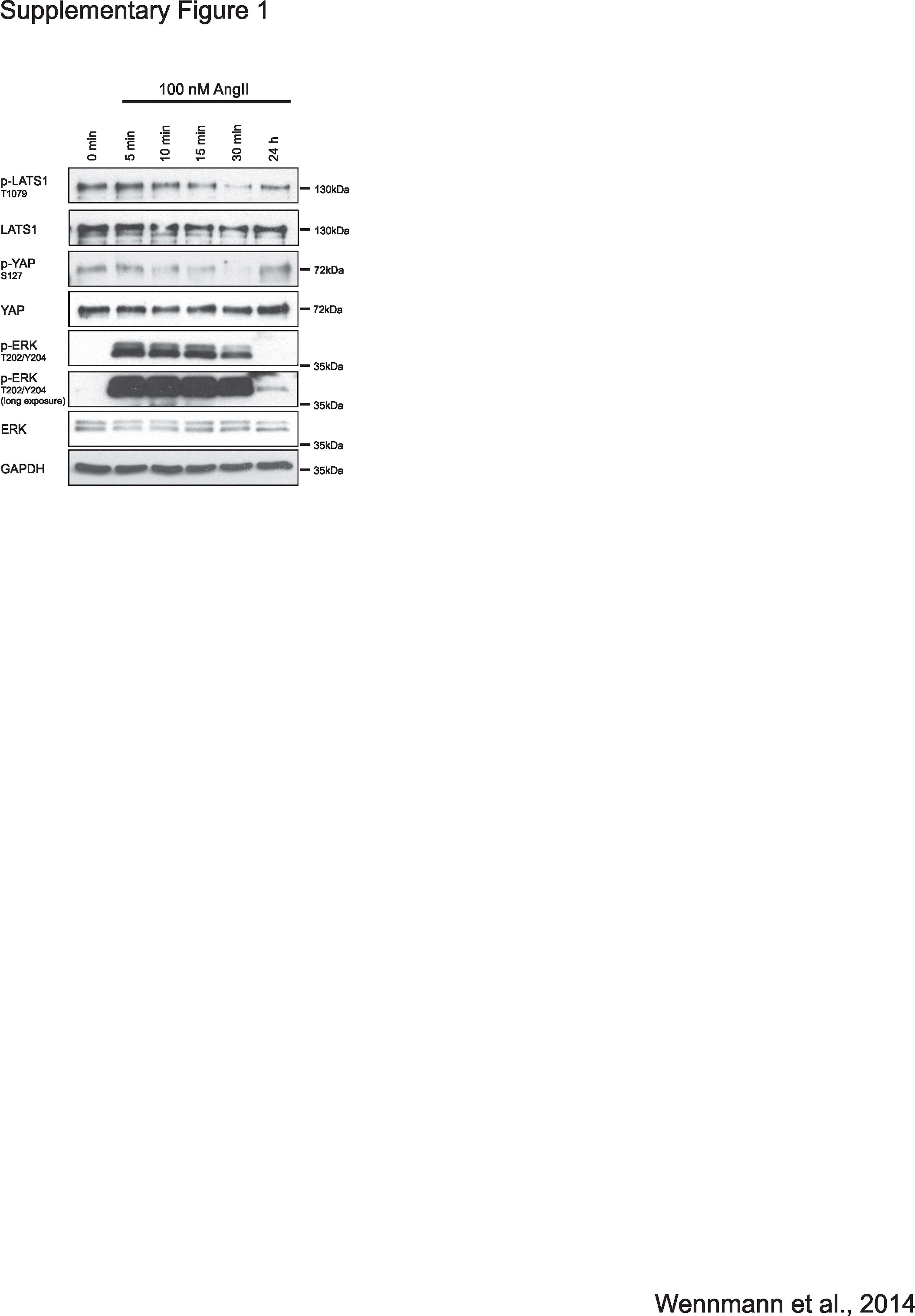

Supplement: Supplementary Figure 1 [file cddis2014476x1.tif]

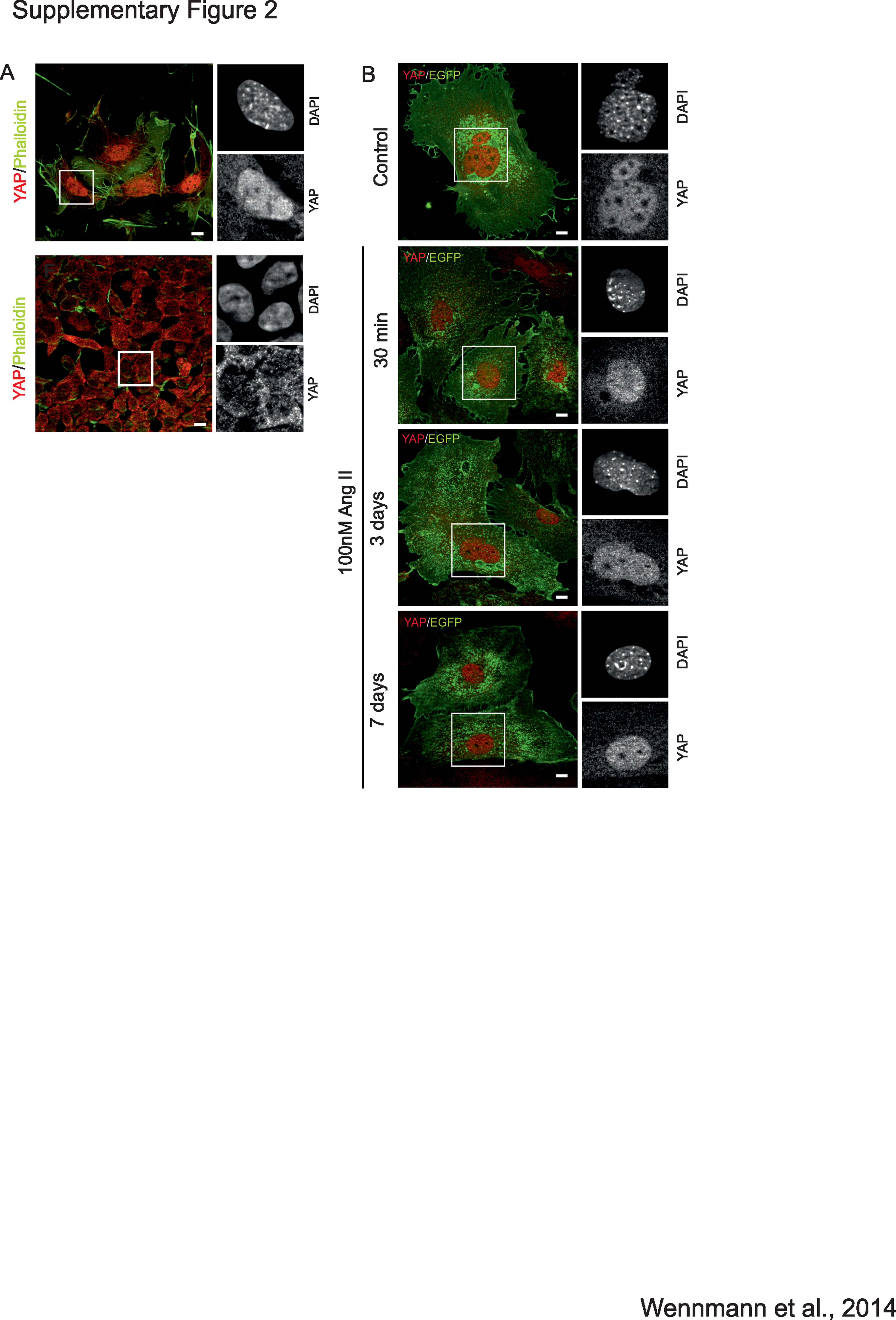

Supplement: Supplementary Figure 2 [file cddis2014476x2.tif]

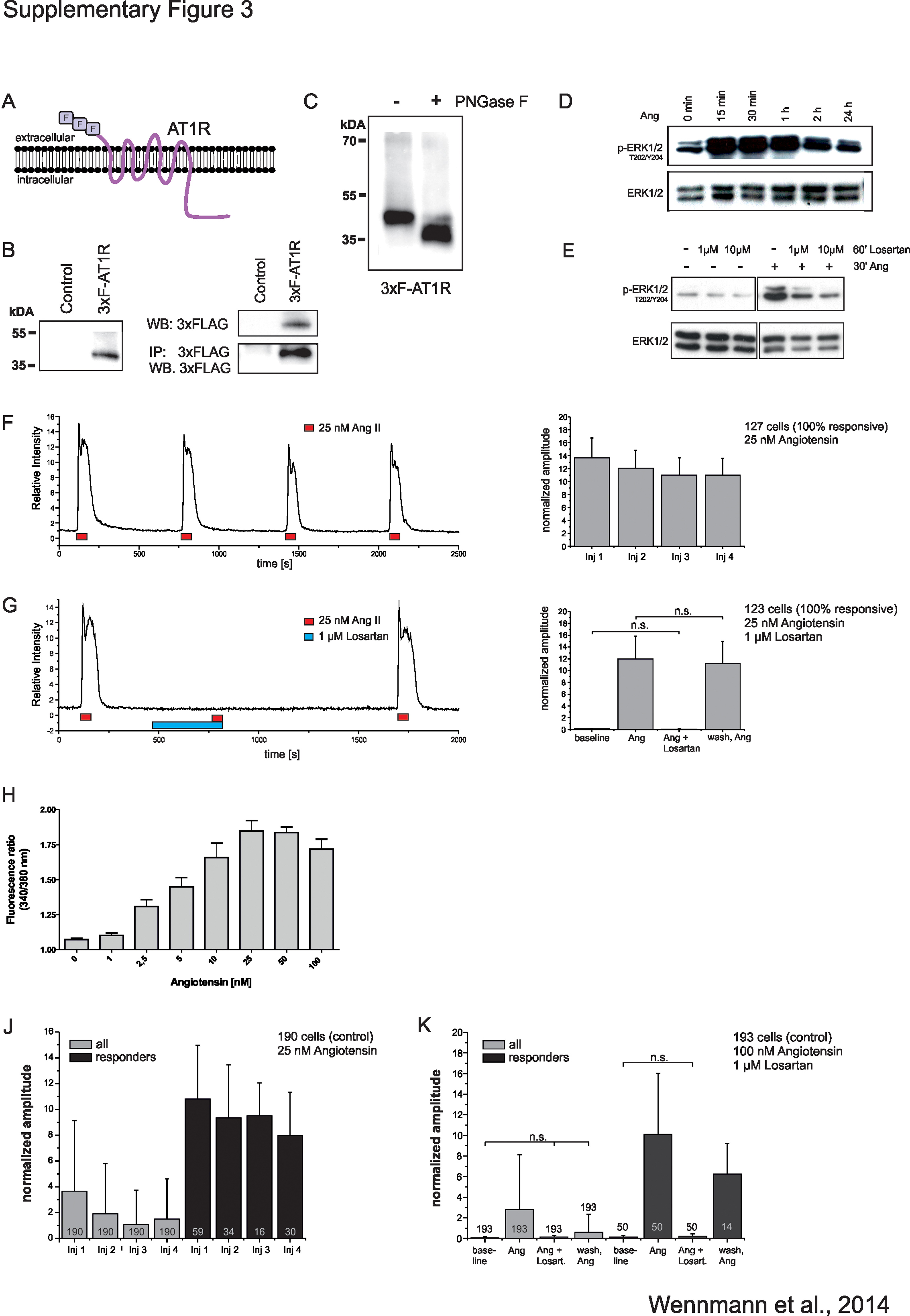

Supplement: Supplementary Figure 3 [file cddis2014476x3.tif]

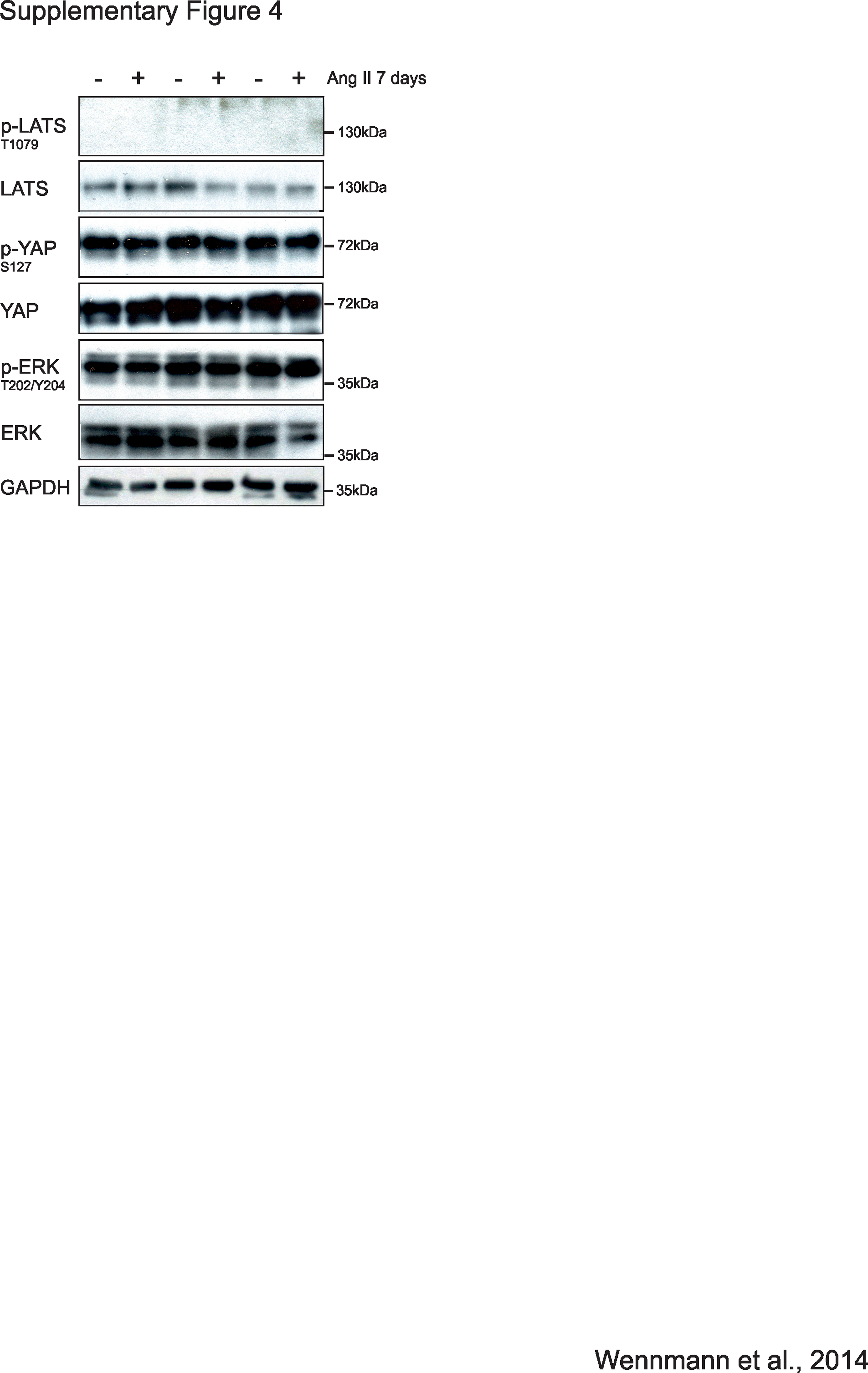

Supplement: Supplementary Figure 4 [file cddis2014476x4.tif]

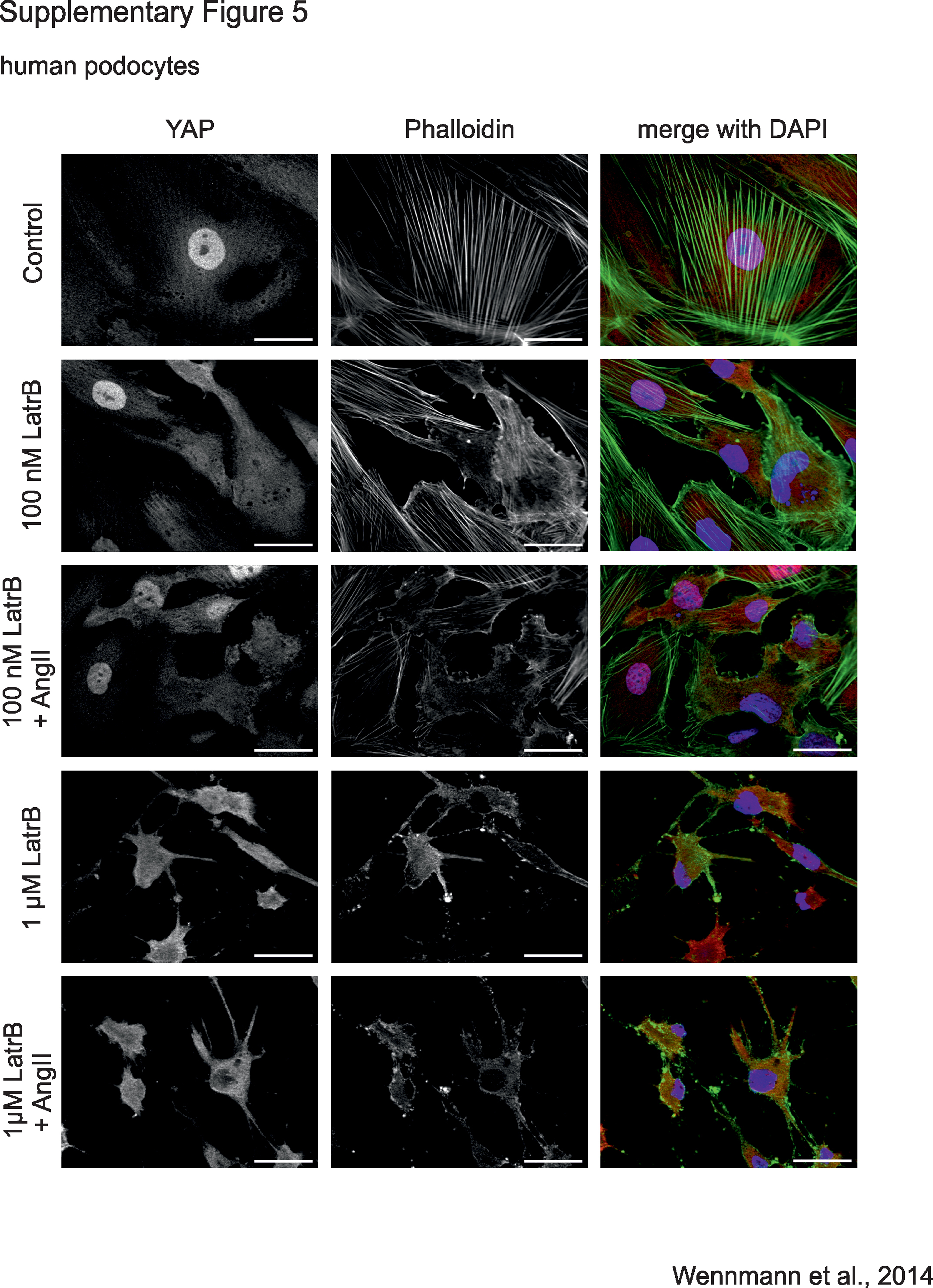

Supplement: Supplementary Figure 5 [file cddis2014476x5.tif]

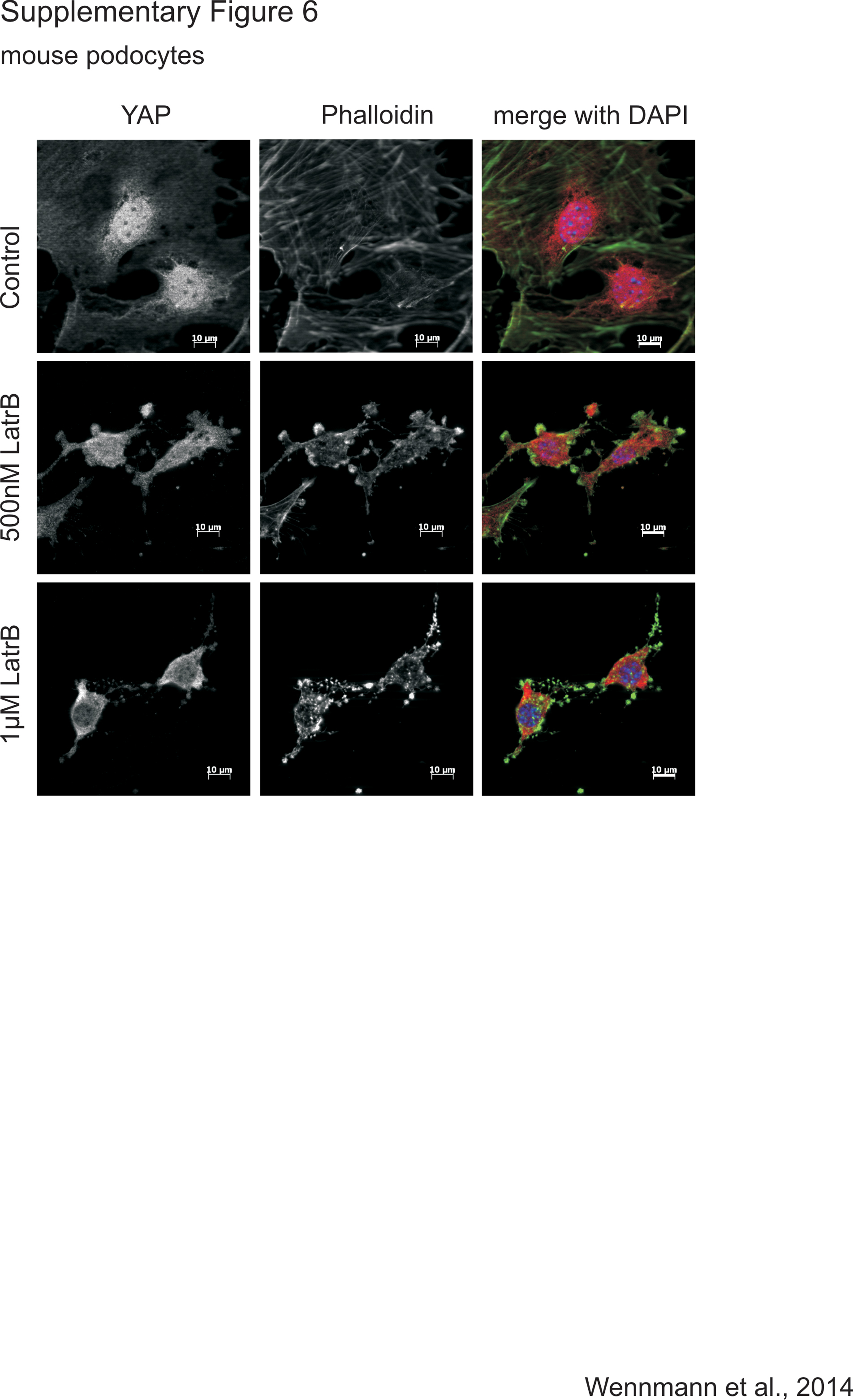

Supplement: Supplementary Figure 6 [file cddis2014476x6.tif]

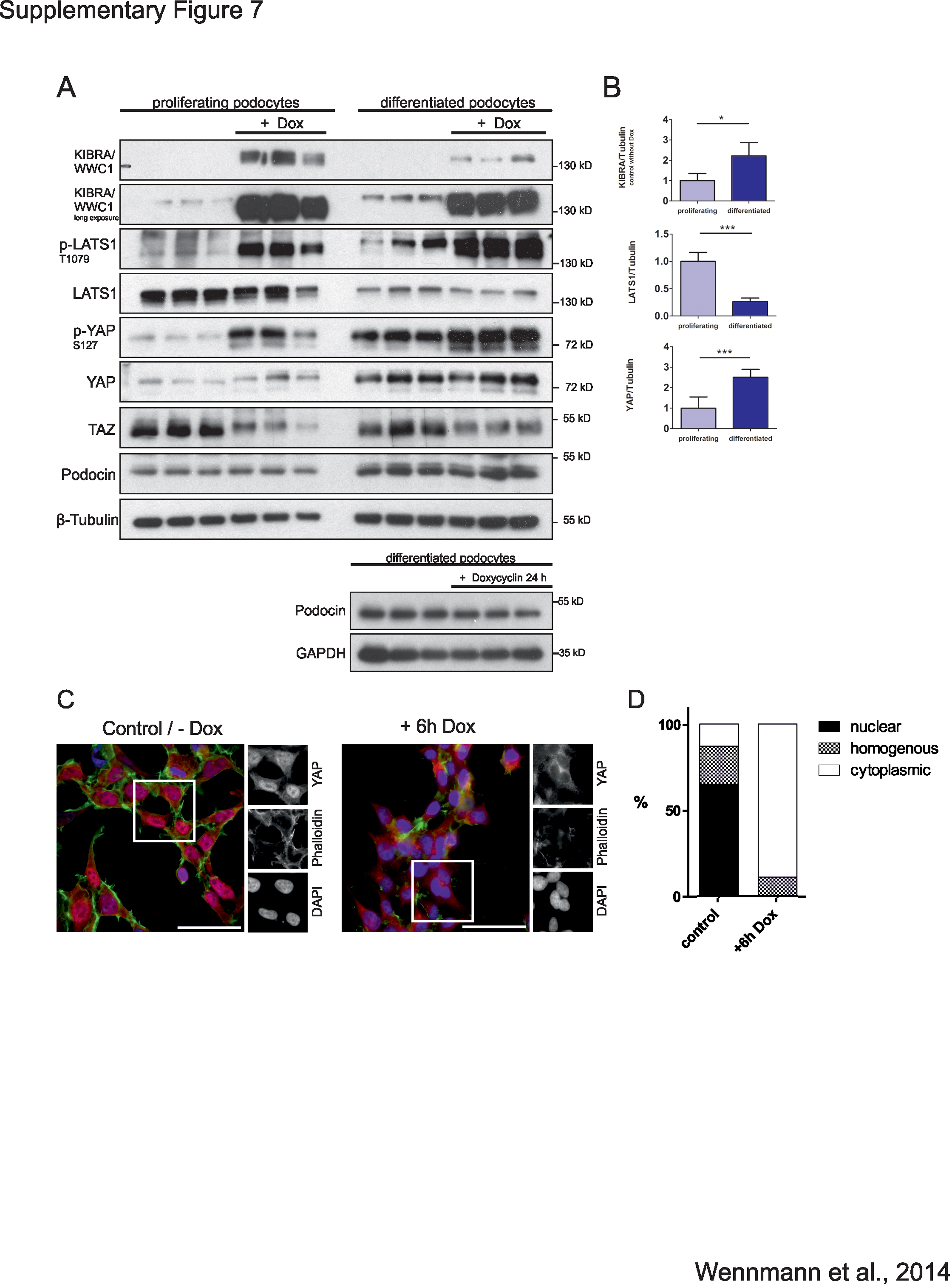

Supplement: Supplementary Figure 7 [file cddis2014476x7.tif]

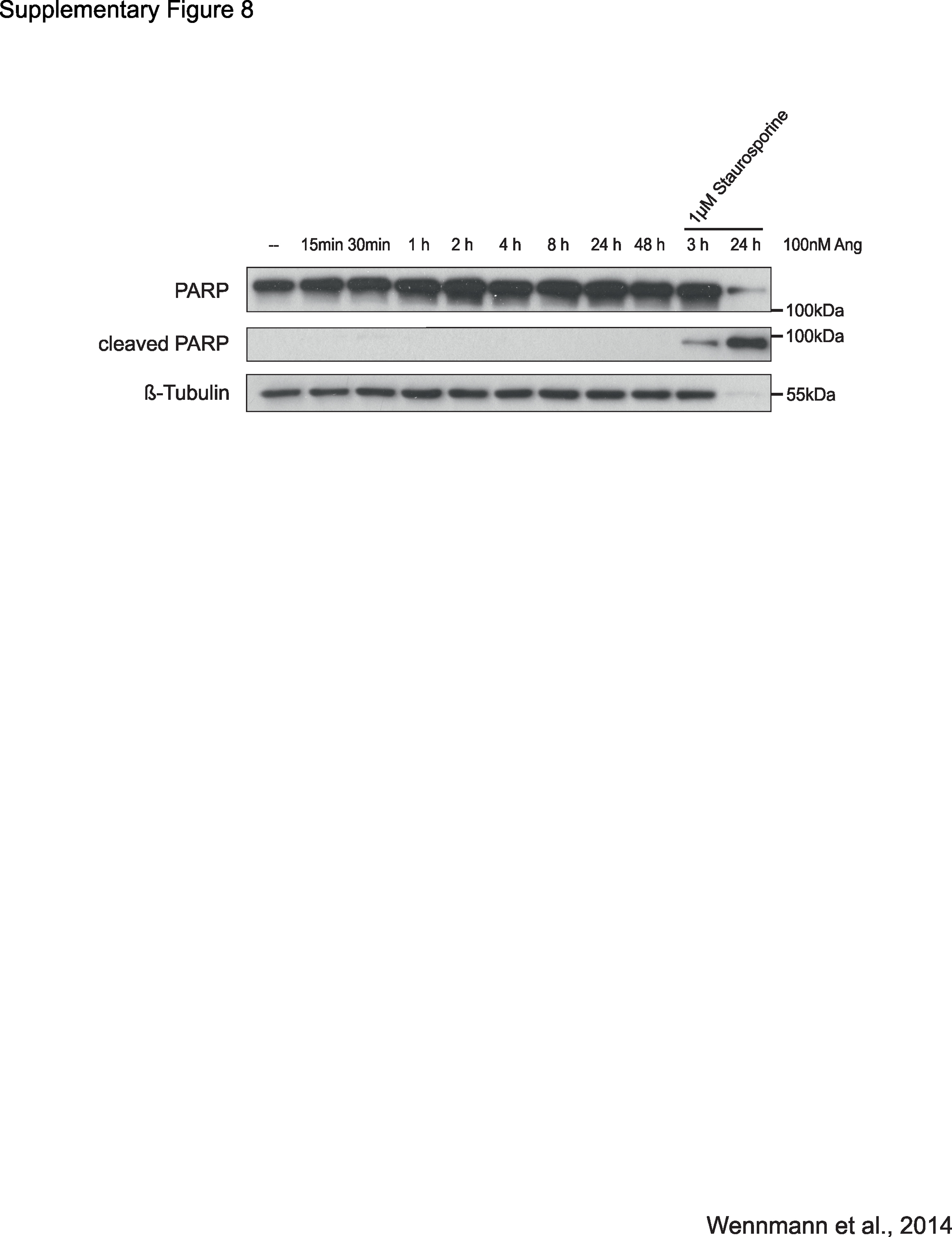

Supplement: Supplementary Figure 8 [file cddis2014476x8.tif]
