# Supplementary material for: The Hippo pathway is controlled by Angiotensin II signaling and its reactivation induces apoptosis in podocytes
Source: Cell Death Dis. 2014 Nov 13;5(11):e1519–. doi: 10.1038/cddis.2014.476 (PMC4260734; doi:10.1038/cddis.2014.476)
Supplement: Supplementary Figure Legends [file cddis2014476x9.doc]

**Supplementary Figures**

**Supplementary Figure 1: Time course of Ang II stimulation inactivating Hippo-signaling**

HEK293 cells stably overexpressing AT1R were stimulated with Ang II for the indicated time period. Cell lysates were used to detect endogenous LATS1, p-T1079-LATS1, YAP, p-S127-YAP, ERK1/2, p-T202/Y204-ERK1/2. Endogenous GAPDH served as a loading control.

**Supplementary Figure 2: YAP localization in mouse podocytes and HEK293T cells**

**A:** Immunofluorescence staining of YAP (red) showed mainly nuclear localization of YAP in mouse podocyte cell line (upper picture). HEK293T cells showed a cell density dependent YAP distribution with a mainly cytoplasmic distribution in near confluent HeK293 cells. Phalloidin (green) marked the actin cytoskeleton. Scale bars represent 10 µm. **B:** Immunofluorescence staining of YAP (red) in isolated genetically marked primary mouse podocytes (EGFP, green) stimulated with 100 nM Ang II for 30 min, 3 days or 7 days showed mainly nuclear localization of YAP even after prolonged Ang II stimulation. Non-stimulated cells served as control.

**Supplementary Figure 3: Characterization of human podocytes stably overexpressing AT1R**

**A:** Schematic overview of the 3xFLAG marked AT1R integrated into the membrane. **B:** Western blot analysis shows human podocytes stable expressing 3xFLAG tagged AT1R using anti-FLAG antibody (left). The 3xFLAG-AT1R could be enriched by immunoprecipitation using agarose beads covalently coupled to anti-FLAG antibody, shown in a Western blot experiment with anti-FLAG antibody (right). **C:** Treatment with PNGase F led to a downshift of the AT1R signal on Western blot showing the posttranslational glycosylation of the overexpressed protein; anti FLAG antibody was used to detect AT1R. **D:** Western Blot experiment using ERK and p-ERK (T202, Y204) antibodies shows the time dependent activation of ERK signaling after Ang II treatment. **E:** Ang II induced ERK activation could be blocked by Losartan. **F:** Calcium imaging with fluorescence emission measurement of AT1R overexpressing podocytes. Stimulation with Ang II (25 nM) led to a reversible increase of the intracellular calcium concentration in all tested cells, which could be induced several times. *Left:* Example for the reaction of one cell. *Right:* Statistical analysis of the calcium influx in all measured cells. 100% of the cells responded to Ang II. **G:** This calcium influx caused by Ang II stimulation could be reversibly blocked by a short pre-incubation with the AT1R agonist Losartan (1 µM). *Left:* Example for the reaction of one cell. *Right:* Statistical analysis of the calcium influx in all measured cells. 100% of the cells responded to Ang II. **H:** The amount of calcium influx depended on Ang II concentration, fluorescence emission measured with a fluorescence plate reader after pre-incubation of the cells with Fura 2-AM. Calcium influx went into saturation with an Ang II concentration of 25 nM. **J:** Statistical analysis of Calcium influx in podocyte cells from the originally established podocyte cell line stimulated with Ang II. The reaction of 190 cells was measured and averaged (grey columns). Only a part of the cells responded to Ang II (small numbers in black columns). The average value of fluorescence emission of responding cells is shown in the black columns. **K:** The calcium influx in wildtype podocytes could also be blocked by a short pre-incubation with the AT1R agonist Losartan (1 µM). Grey columns show the averaged results of all measured cells, black columns those of all cells that responded to Ang II, small numbers indicate the number of evaluated cells, respectively. By contrast to the established stable AT1R overexpressing podocytes (**F**), originally established podocytes (2) only partially responded to Ang II treatment.

**Supplementary Figure 4: Longtime treatment of podocytes with Ang II do not alter Hippo pathway activity**

Western blot experiment with extracts from podocytes overexpressing AT1R stimulated with Ang II for 7 days using the indicated antibodies showed that also longtime treatment with Ang II had no influence on Hippo pathway in podocytes. GAPDH served as loading control.

**Supplementary Figure 5: Disruption of the actin cytoskeleton with Latrunculin B leads to cytoplasmic YAP distribution in differentiated human podocytes**

Immunofluorescence staining of endogenous YAP (red) in human podocytes, differentiated for 10 days, treated with Latrunculin B in the indicated concentrations for 10 min before stimulation with Ang II (100 nM) for further 30 min. The disruption of the actin cytoskeleton (Phalloidin, green) led to a beginning of cytoplasmic YAP distribution in cells treated with 100 nM Latrunculin B. These cells also show a beginning of depolarization and breakup of the actin fibers. Treatment with 1 µM Latrunculin B led to a more distinct disruption of the actin cytoskeleton with a clear cytoplasmic YAP distribution. The YAP shuttling could not be restored by subsequent Ang II stimulation. Scale bars represent 50 µm.

**Supplementary Figure 6: Disruption of the actin cytoskeleton with Latrunculin B leads to cytoplasmic YAP distribution in a mouse podocyte cell line**

Immunofluorescence staining of endogenous YAP (red) in mouse podocytes, treated with Latrunculin B in the indicated concentrations for 40 min showed that disruption of the actin cytoskeleton (Phalloidin, green) led to cytoplasmic distribution of YAP. The nuclei are marked with DAPI. Scale bars represent 10 µm.

**Supplementary Figure 7: Re-activation of Hippo signaling by induction of KIBRA/WWC1 increases the phosphorylation of LATS kinase and YAP in proliferating and differentiated podocytes**

**A:** Indirect activation of LATS1 kinase due to inducible KIBRA/WWC1 overexpression (doxycycline treatment, 125 ng/ml for 5 h) was used to verify the link between LATS activation and nuclear export of YAP. Cell lysates of doxycycline treated and untreated cells were utilized to detect endogenous LATS1, p-1079-LATS1, YAP, p-S127-YAP, KIBRA/WWC1, TAZ, Podocin and β-Tubulin, which served as loading control. Overexpression of KIBRA/WWC1 in podocytes (proliferating podocytes left side and differentiated cells right side) resulted in a robust increase in LATS und YAP phosphorylation. LATS expression was reduced in differentiated podocytes whereas YAP expression was increased. Interestingly, we could observe an increase of phosphorylation at T1079-Lats in differentiated podocytes compared with proliferating podocytes which showed only a weak phospho-signal at T1079. KIBRA/WWC1 expression was increased in differentiated podocytes. TAZ is also expressed in podocytes. The TAZ signal shifts up like the YAP signal, most probably to a LATS dependent phosphorylation, when KIBRA/WWC1 is expressed. Podocin expression was increased as expected in differentiated podocytes. No change in Podocin expression was detectable after induction of KIBRA expression after 6 and 24 hours. **B:** The ratio between the protein of interest and β-Tubulin is calculated and indicated (Mean and SD, t-Test,*p<0.05, ***p<0.001). **C:** Immunofluorescence analysis of endogenous YAP (red) in subconfluent HEK293T cells showed enhanced cytoplasmic YAP distribution after overexpression of KIBRA/WWC1 by induction with doxycycline (125 ng/ml) for 5 hours. **C:** Statistical analysis of 100 cells from the experiment shown in B.

**Supplementary Figure 8: Angiotensin II stimulation does not induce apoptosis in AT1R overexpressing human podocytes**

Cell extracts from AT1R overexpressing podocytes treated with 100 nM Ang II over a time course were used in Western Blot experiments to detect endogenous PARP and cleaved PARP. β-Tubulin served as loading control. For a positive control we treated the cells with 1 µM Staurosporine for 3 and 24 hours. We could not detect an increase of cleaved PARP after stimulation with Ang II but we could detect a strong cleaved PARP signal after Staurosporine treatment.

**Supplementary Reference List**

Reference List

(1) Schneider CA, Rasband WS, Eliceiri KW. NIH Image to ImageJ: 25 years of image analysis. Nat Methods 2012 Jul;9(7):671-5.

(2) Saleem MA, O'Hare MJ, Reiser J, Coward RJ, Inward CD, Farren T, et al. A conditionally immortalized human podocyte cell line demonstrating nephrin and podocin expression. J Am Soc Nephrol 2002 Mar;13(3):630-8.
